# The intracellular virus-host interface of henipaviruses

**DOI:** 10.1128/jvi.00770-25

**Published:** 2025-07-18

**Authors:** Melanie N. Tripp, Stephen M. Rawlinson, Sarah J. Edwards, Jasmina M. Luczo, Glenn A. Marsh, Kim Halpin, Gregory W. Moseley

**Affiliations:** 1 Department of Microbiology, Biomedicine Discovery Institute, Monash University 161661, Clayton, Victoria, Australia; 2 Australian Centre for Disease Preparedness, Commonwealth Scientific and Industrial Research Organisation (CSIRO), East Geelong, Victoria, Australia; Indiana University Bloomington, Bloomington, Indiana, USA

**Keywords:** henipavirus, virus-host interface, Hendra virus, Nipah virus, viral proteins, immune evasion

## Abstract

The *Henipavirus* genus comprises five viral species, of which the prototype members, Hendra virus (HeV) and Nipah virus (NiV), are reported to infect humans. In humans and other spill-over hosts, HeV/NiV can cause severe respiratory and/or encephalitic disease, with mortality rates exceeding 50%; currently, there are no approved human vaccines and only limited therapeutic options. As members of the family *Paramyxoviridae*, henipaviruses have six “core” structural proteins and typically three additional accessory proteins that are expressed from the P gene. Several of these proteins are multifunctional, with roles in forming intracellular interfaces with the host (in particular, M, P, V, W, and C proteins), to modulate processes including antiviral responses, supporting viral replication. Understanding the molecular basis of these interfaces and their functions is critical to delineate the mechanisms of pathogenesis and may inform new strategies to combat infection and disease. Recent research has significantly advanced the understanding of the functions and interactions of multifunctional intracellular henipavirus proteins, including revealing novel roles in subverting the nucleolar DNA damage response (DDR) and modulating the functions of 14-3-3 proteins. This review will discuss the intracellular virus-host interface, focusing on the M, P, V, W, and C proteins of HeV/NiV, with a focus on recently identified functions and interactions.

## INTRODUCTION

**Fig 1:**
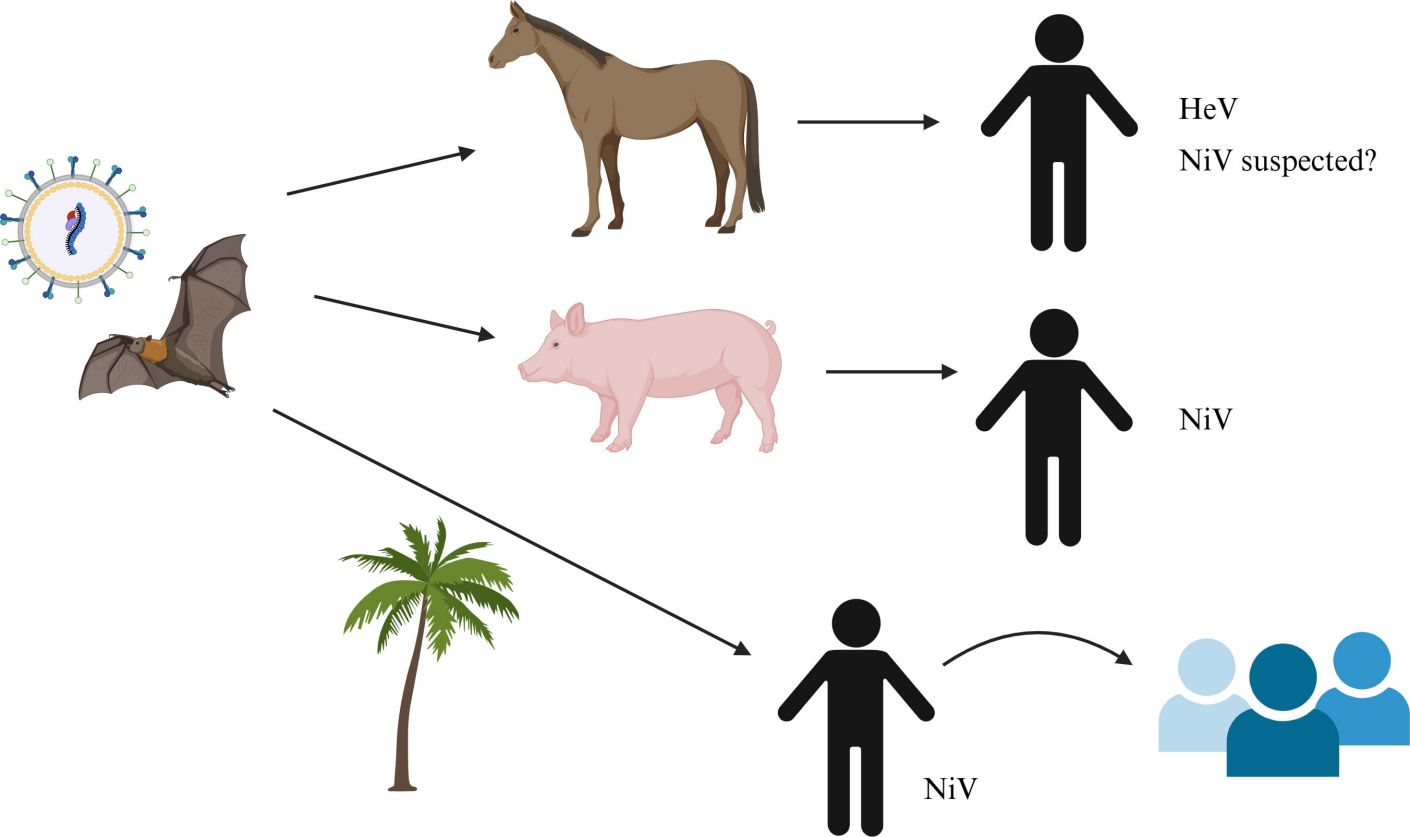
Transmission patterns of HeV and NiV. HeV has spilled over from fruit bats to horses, with some cases leading to human infections by HeV genotype 1 (3). NiV-Malaysia infected humans following spillover to pigs during an outbreak in Malaysia and Singapore. NiV-Bangladesh has infected humans via the consumption of date palm sap/fruit, likely contaminated by infected bats before further spreading via human-to-human transmission (2). A suspected NiV outbreak in the Philippines involved horse-to-human transmission, followed by human-to-human transmission (8).

Henipaviruses are pleomorphic, enveloped, non-segmented negative-stranded RNA viruses (order *Mononegavirales*, family *Paramyxoviridae*, subfamily *Orthoparamyxovirinae*) (1). The henipavirus genus was identified in the 1990s and includes the highly pathogenic Hendra (HeV) and Nipah viruses (NiV), as well as Cedar (CedV), Ghana (GhV), and Angavokely viruses (AngV) (1). HeV and NiV each comprise at least two genotypes, HeV genotype 1 and genotype 2, and NiV Malaysia and NiV Bangladesh. The reservoir hosts of henipaviruses are fruit bats in the family *Pteropodidae*, but HeV and NiV can infect a range of mammals (including humans), causing disease with high case-fatality rates and similar respiratory and neurological pathologies (2, 3). CedV, GhV, and AngV are not as extensively researched as HeV and NiV and are not reported to cause human infection. Their host range is also less well-defined, although CedV and GhV use similar entry pathways to HeV/NiV (via ephrin receptors) (4–6), whereas AngV utilizes a different, as-yet-unidentified receptor (7). There have been seven recorded HeV human infections (resulting in four deaths), all of which occurred through contact with infected horses, presumed to follow bat-horse transmission, and all due to HeV genotype 1 (3). NiV has caused hundreds of human infections and deaths (involving both the Malaysia and Bangladesh genotypes), with more complex transmission patterns, including bat-to-human and human-to-human transmission (2) (Fig. 1).

HeV/NiV are classified as risk group 4 pathogens, with NiV and other henipaviral diseases designated a WHO priority disease due to epidemic potential and lack of substantial preventative or therapeutic medical countermeasures (9). Although there are no approved vaccines for humans, a licensed vaccine for horses based on a soluble HeV G protein is available (10). Additionally, there are other F/G protein-based vaccines and monoclonal antibodies (mAbs) in clinical trials (11). However, high costs associated with mAbs (mAb treatment for SARS-CoV-2, for example, can exceed $2000 [12]) may limit usefulness in low- and middle-income countries where NiV outbreaks are occurring (2). Thus, alternative strategies are required, potentially including small molecule inhibitors of intracellular targets/interfaces.

In June 2024, the International Committee on Taxonomy of Viruses created a new genus, *Parahenipaviruses*, the members of which were previously considered to be henipaviruses (13). This includes more recently identified henipa-like viruses with non-bat reservoirs, which appear not to use ephrin receptors and may be characterized by an additional open-reading frame (ORF) in the genome (14–18). Many parahenipaviruses are identified only as partial or full genome sequences, with no virus isolate obtained (reviewed in [3, 14, 15, 19]). Langya virus (*Parahenipavirus langyaense*) has been implicated in human infection with no associated mortality events (20), and Mojiang virus (*Parahenipavirus mojiangense,* MojV) was suspected of causing severe pneumonia resulting in three fatalities (21), although it was not conclusively identified as the etiological agent. No other parahenipavirus has been reported to infect humans, and therefore, the potential risk that parahenipaviruses and henipaviruses other than HeV/NiV may pose is still unclear (14). It is also possible that AngV may be distinct from the “classical” henipavirus genus, based on differing receptor usage and phylogenetic analysis (14), although it shares the same reservoir species (pteropodid bat) (7). Several other recent reviews discuss the nature, epidemiology, and phylogenetic relationship of henipaviruses/parahenipaviruses; this review aims to address the intracellular molecular interactions and functions of henipavirus proteins, in particular, in the modulation of the host cell, and hence, we will focus on the highly pathogenic HeV and NiV, which are the best studied in this respect. Compared with virus-cell surface receptor interactions, the intracellular virus-host interface of HeV and NiV has been relatively poorly studied in terms of potential therapeutic targeting, despite having roles in many important aspects of the virus life cycle, including immune evasion. A greater understanding of the intracellular virus-host interface will both reveal the molecular events underlying henipaviral diseases and potentially enable the identification of new targets for antiviral strategies.

## HENIPAVIRUS GENOME STRUCTURE

**Fig 2:**
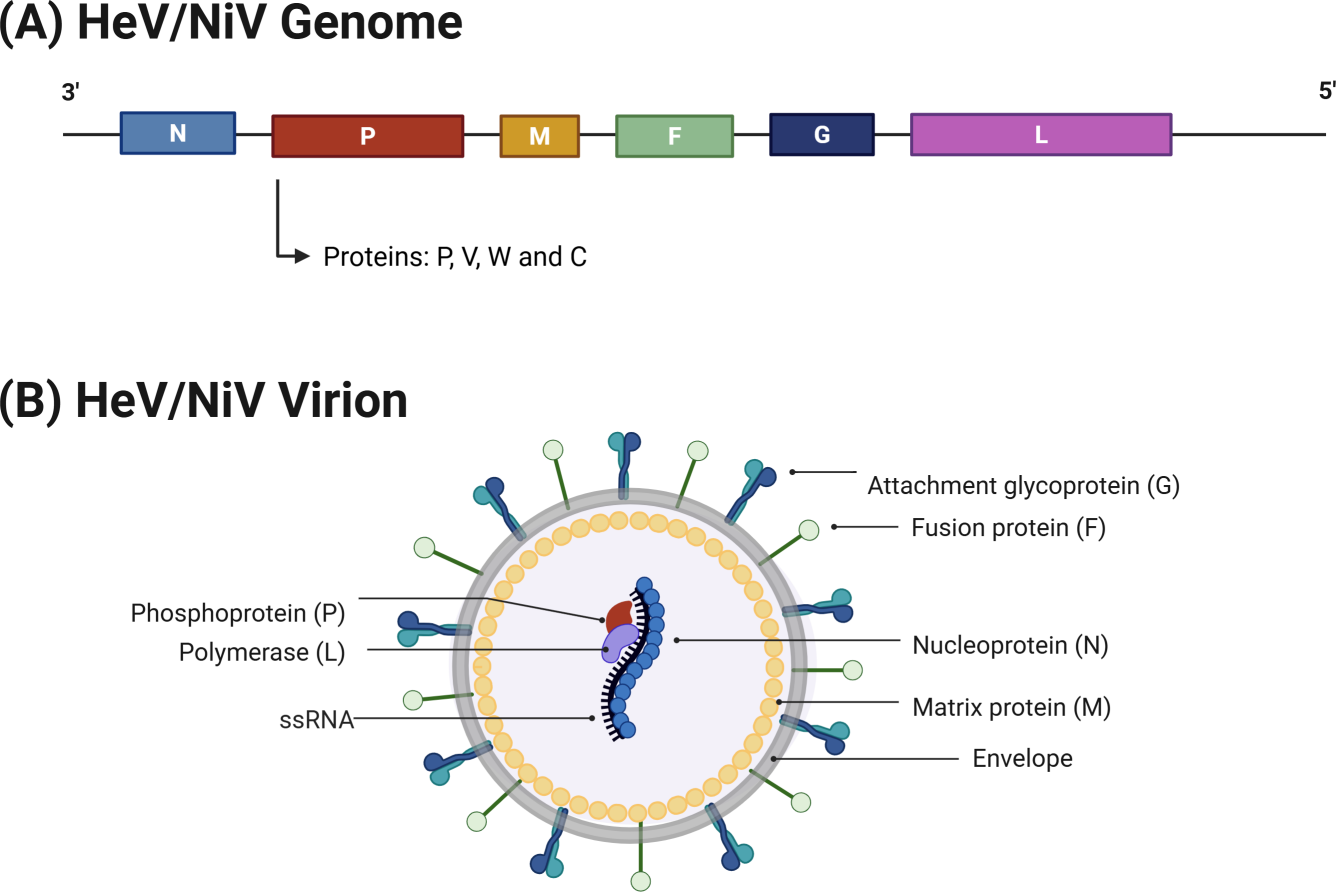
HeV/NiV genome and virion structure. (**A**) The HeV/NiV genome contains six genes (from 3’ to 5’: N, P, M, F, G, L; P encodes P protein and V, W, and C accessory proteins) (22). (**B**) N protein associates with the single-stranded RNA genome (ssRNA) to form the nucleocapsid; P protein is a cofactor of L protein, the catalytic component of the RdRp; together, P and L constitute the functional RdRp complex. M protein associates with the inner leaflet of the envelope and is critical for virion budding (22). G and F proteins are transmembrane proteins embedded in the envelope that mediate receptor binding and promote fusion with cellular membranes, respectively. HeV/NiV proteins also have various host cell interactions/modulatory roles, and several can traffic to different cellular compartments including the nucleus (M, V, W, and C proteins) and nucleolus (M protein) (see text).

Paramyxoviruses are negative-sense RNA viruses with genomes of 14.6–20.1 kb, containing six “core” genes that are largely conserved across the family. These encode for six structural proteins: from 3’−5’, N (encoding nucleocapsid protein), P (encoding phosphoprotein), M (matrix), F (fusion), RBP (receptor binding protein, designated G [glycoprotein] for henipaviruses, H or HN protein in other paramyxoviruses), and L (‘large’, the RNA-dependent RNA polymerase [RdRp]). Furthermore, several accessory proteins are typically encoded within the P gene in addition to the conserved essential polymerase cofactor P protein (Fig. 2) (22). Accessory proteins generally encoded in the henipavirus P gene include V and W proteins, which are expressed via an RNA-editing mechanism, and C protein(s), which are encoded in alternate ORFs. The accessory proteins can have highly distinct properties, including in subcellular localization and molecular interactions, which confer unique functions that presumably account for the evolutionary overprinting of several proteins in the P gene. HeV P is also hypothesized to encode the small basic (SB) protein via an alternate ORF (23). P genes of other paramyxovirus genera similarly encode multiple proteins; most encode a cysteine-rich V protein and one or more C proteins in alternative ORFs (22). The N, P, M, F, G, and L proteins have conserved roles in virion structure and replication (Fig. 2); however, several of these proteins, along with the accessory proteins, have functions in modulating host cell biology.

## HENIPAVIRUS INTERACTIONS WITH THE TYPE I INTERFERON RESPONSE

**Fig 3:**
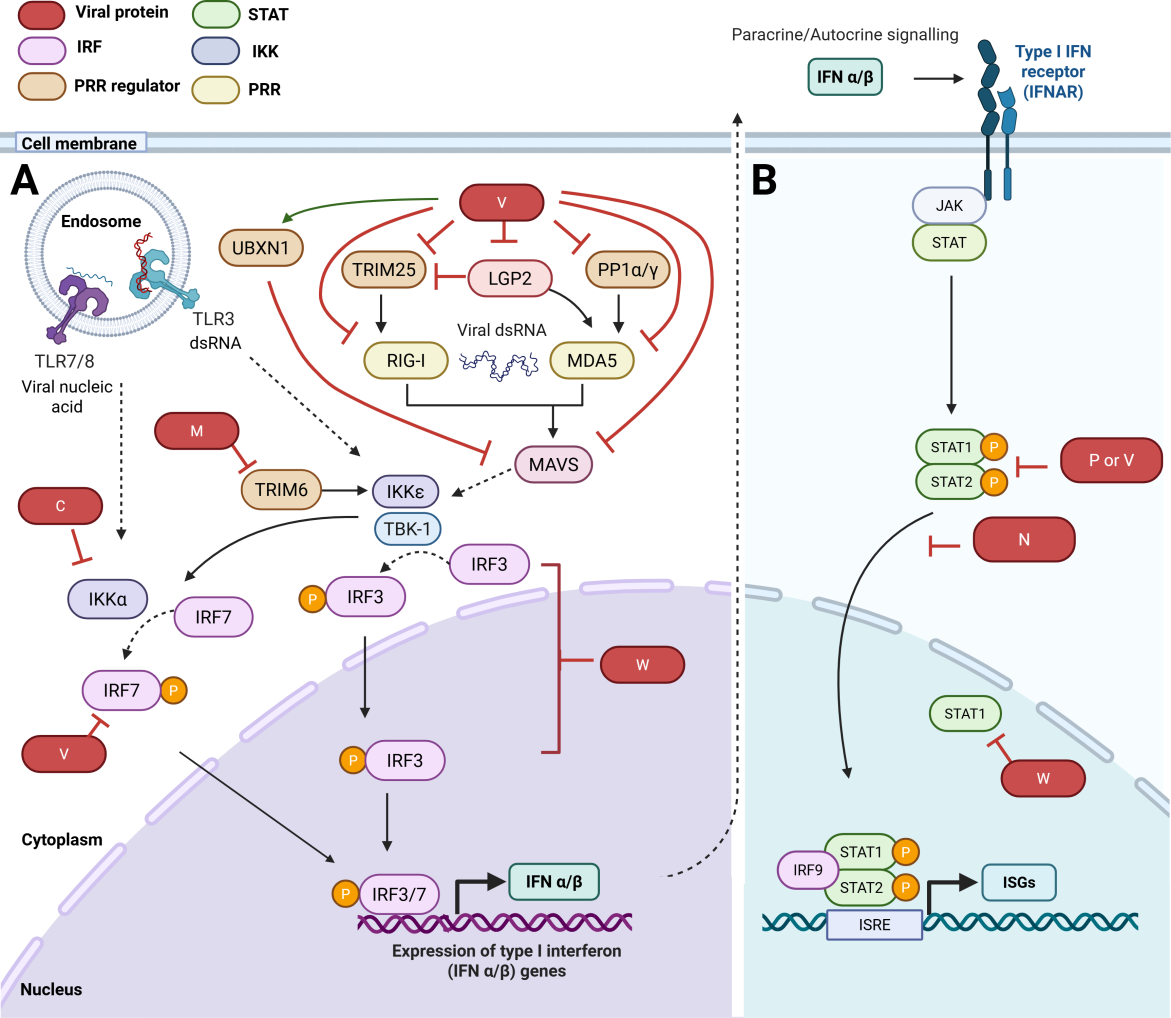
Type I IFN response to infection by RNA viruses and antagonism by henipavirus proteins. (**A**) IFN induction: following infection, viral RNA products (e.g., double-stranded (dsRNA), uncapped RNA) are detected by pattern-recognition receptors (PRRs), including RIG-I like receptors (RLRs; cytoplasmic receptors including retinoic acid–inducible gene I [RIG-I], melanoma differentiation-associated protein 5 [MDA5], and laboratory of Genetics and Physiology 2 [LGP2]) and Toll-like receptors (TLRs; transmembrane receptors that include endosomal TLR3 and TLR7/8) (26). RLRs are regulated by proteins including Tripartite motif 25 (TRIM25) for RIG-I; Phosphatase 1 α/γ (PP1α/γ) for MDA5; and LGP2, for both RIG-I and MDA5 (32–35). Activated PRRs interact with adapter proteins (e.g., RIG-I/MDA5 with MAVS; TLR7/8 with MyD88; TLR3 with TRIF [not shown, dashed lines]) (26), regulators of which include UBX domain-containing protein 1 (UBXN1) for MAVS (36). These adaptors recruit and activate signaling factors including kinases (e.g., Inhibitor of κB kinases [IKKs] and TANK binding kinase 1 [TBK1]), leading to phosphorylation of transcription factors including IFN-regulatory factors (IRFs) 3 and 7, and nuclear factor κB (not shown). IRFs enter the nucleus and bind to promoter regions to induce antiviral genes, including Type I IFNs (26). Viral antagonism: NiV V protein directly binds and inhibits RIG-I/MDA5 and regulates TRIM25/PP1α/γ/LGP2 to inhibit RIG-I/MDA5 activation (33, 34, 37, 38). V protein also stabilizes UBXN1 (which inhibits MAVS [36]), directly targets MAVS for degradation (39), and binds and inhibits IRF7 (40). C protein interacts with and inhibits IKKα (preventing IKKα-mediated phosphorylation/activation of IRF7) (41). W protein inhibits TBK1- and IKKε-dependent IRF3 phosphorylation (42). M protein inhibits TRIM6 (ubiquitin ligase regulator of IKKε), thereby suppressing IRF3 activation (43). (**B**) IFN signaling: Following production and release from cells, Type I IFN signals in autocrine or paracrine fashion by binding to the Type I IFN receptor (IFNAR), which activates receptor-associated Janus kinases (JAKs) that phosphorylate STAT1 (Signal Transducers and Activators of Transcription 1) and STAT2 at conserved tyrosine residues. Activated phospho-STAT1/2 dimerize, translocate to the nucleus, and, in a complex with IRF9, bind to genomic IFN-stimulated response elements (ISREs) to regulate the transcription of IFN-stimulated genes (ISGs) (44). Viral antagonism: P, V, W, and N proteins inhibit STAT1/2 responses (45, 46).

The type-I interferon (IFN) system mediates the primary antiviral response of host cells and can be directly activated by RNA and DNA sensors, which detect viral infections via direct recognition of viral components or replication products (Fig. 3) as well as via virus-induced damage to cellular organelles (24–26). This leads to the induction of IFN, which is released from cells and acts on the infected and neighboring cells to induce IFN signaling (Fig. 3), which is critical to the establishment of an innate cellular antiviral state; type-I IFN also contributes to the development of an adaptive response (immunological landscape of NiV infection reviewed recently [27]). Viral mechanisms to antagonize the IFN system and subvert immune responses are thus essential to infection and pathogenesis and are among the best characterized intracellular virus-host interfaces (28–30). Many viruses, including henipaviruses, express proteins able to target and interfere with both IFN induction and subsequent IFN-dependent signaling (Table 1; Fig. 3) (28, 29, 31).

**TABLE 1:** Characterized intracellular protein interactions of HeV or NiV proteins implicated in modulation of host cell biology

Viral protein	Host protein interaction^*a*^	Function	Henipavirus species reported	Reported for other paramyxoviruses?
N	STAT1 and STAT2	Inhibits IFN-activated nuclear import of STAT1/2, suppressing IFN signaling	HeV and NiV (45)	Yes, measles virus (47) and mumps virus nucleocapsid protein (48)
M	AP-3 complexes	Involved in VLP production, suggesting potential roles in virion formation	HeV and NiV (49)	Currently no reports
	ANP32B	Unknown function; possibly involved in nuclear localization of M protein and virus replication	HeV and NiV (50)	Yes, Newcastle disease virus (NDV), Sendai virus (SeV), and measles virus (51)
	Exportin 1 (XPO1)	Nucleocytoplasmic trafficking (nuclear export)	HeV and NiV (52, 53)	Yes, respiratory syncytial virus (RSV) (54), and although no direct interactions are reported for other paramyxovirus M proteins, several are found in both the nucleus and cytoplasm (53, 55)
	Fibrillarin (FBL)	Implicated in the synthesis of viral RNA and protein	HeV and NiV (56)	Yes, mumps virus, measles virus, and RSV (56)
	Importin alpha (IMPα)	Nucleocytoplasmic trafficking (nuclear import)	HeV and NiV (52, 53, 57)	No direct interactions reported, but other paramyxovirus M proteins are found in the nucleus and cytoplasm (53, 55)
	TRIM6	Inhibits Type I IFN induction *via* IKKε/TBK1	HeV, NiV, and other henipaviruses (GhV and CedV) (43)	Currently no reports
	Treacle	Inhibits rRNA biogenesis; potential role in immune evasion, but specific function not defined	HeV, NiV, and CedV (58, 59)	Yes, MojV and mumps virus (59, 60)
	RAD18	RAD6A (E2 ubiquitin-conjugating enzyme) ubiquitinates M protein residue K258 *via* direct interaction with RAD18, roles in nucleocytoplasmic trafficking	HeV and NiV (61)	Currently no reports
F	Cortactin	Cortactin overexpression inhibits infection by pseudovirus; unknown function/mechanism	NiV (62)	Currently no reports
G	Cortactin	Cortactin overexpression inhibits infection by pseudovirus; unknown function/mechanism	NiV (62)	Currently no reports
P	Polo-like kinase 1 (PLK1)	PLK1 is involved with a range of cellular functions including cell cycle regulation and DNA damage, but the functional outcome is currently unknown	NiV (63)	Yes, parainfluenza virus 5 (PIV5) (64)
	STAT1	Inhibits Type I IFN signaling	HeV and NiV (46, 65–67)	Yes, rinderpest virus (68), measles virus (69) and peste des petits ruminants virus (70)
	STAT4	Inhibits STAT4 activity measured by reporter gene assay; unknown functional outcome	NiV (71)	Currently no reports
V	DNA damage binding protein 1 (DDB1)	DDB1 is part of a ubiquitin ligase complex; unknown functional outcome for HeV/NiV	HeV and NiV (72)	Yes, e.g. PIV5, human parainfluenza virus type 2 (hPIV2) and mumps virus (73)
	Exportin 1 (XPO1)	Nucleocytoplasmic trafficking (nuclear export)	HeV and NiV (66, 74)	No direct interactions reported, but other paramyxovirus V proteins are found in the nucleus and cytoplasm (see review [55])
	Unknown target leading to inhibition of IKKε-induced IRF3 activation	Reduces IFN induction in response to IKKε, but does not significantly reduce IRF3 activation by TLR3	NiV (42)	Currently no reports
	IMPα1	Nucleocytoplasmic trafficking (nuclear import) (74)	HeV and NiV (74)	No direct interactions reported, but other paramyxovirus V proteins are found in the nucleus and cytoplasm (see review [55])
	IRF7	Blocks TLR7/9-dependent signaling	NiV (40)	Yes, e.g., hPIV2, SeV and measles virus (40)
	LGP2	Inhibits activation of MDA5 signaling to reduce Type I IFN induction	HeV and NiV (37, 75)	Yes, e.g., SeV, Menangle virus and Tioman virus (75)
	Targets MAVS for degradation	Inhibits IFN induction	HeV and NiV (39)	Yes, e.g., NDV, SeV and measles virus (39)
	MDA5	Inhibits MDA5 signaling to antagonize Type I IFN induction	HeV and NiV (38, 76)	Yes, e.g., PIV5, hPIV2, and mumps virus (38)
	Nod-like receptor protein, pyrin domain–containing-3 (NLRP3)	Inhibits NLRP3 mediated interleukin 1β (IL-1β) release	NiV (77)	Yes, e.g. SeV, hPIV2, and measles virus (77–79)
	PLK1	PLK1 is involved in cell cycle regulation and checkpoint responses to DNA damage; PLK1 phosphorylates V protein, but the functional outcome is currently unknown	HeV and NiV (63)	Currently no reports
	PP1α/γ	Prevents PP1-mediated dephosphorylation of MDA5 and consequently MDA5 activation	NiV (34)	Yes, measles virus (34)
	RIG-I/TRIM25	Inhibits RIG-I signaling to antagonize Type I IFN induction	NiV (33)	Yes, measles virus, SeV and PIV5 (33)
	STAT1 and STAT2	Inhibit Type I IFN signaling	HeV and NiV (46, 65–67)	Yes, e.g., PIV5 (80), canine distemper virus (81) and rinderpest virus (68)
	STAT4	Inhibits STAT4 activity in reporter gene assay; unknown functional outcome	NiV (71)	Currently no reports
	STAT5	Inhibits STAT5 activity in a reporter gene assay; unknown functional outcome	NiV (71)	Currently no reports
	Interacts with and stabilizes UBXN1	Increases inhibitory function of UBXN1 toward MAVS to reduce Type I IFN induction	NiV (36)	Currently no reports
W	14-3-3 family proteins	Range of effects including downregulation of immune genes and NF-κB inflammatory pathway	HeV and NiV (82, 83)	Currently no reports
	Exportin 1 (XPO1)	Nucleocytoplasmic trafficking (nuclear export)	HeV and NiV (74, 84)	No direct interactions reported, but several avian paramyxovirus W proteins are predicted to contain a NES (85)
	IMPα1 and IMPα3	Nucleocytoplasmic trafficking (nuclear import)	HeV and NiV (84, 86)	No direct interactions reported, but several avian paramyxovirus W proteins are predicted to contain a NLS (85)
	Unknown target leading to inhibition of phosphorylation of IRF3	Inhibits induction of IFN-β	NiV (42)	Currently no reports
	PLK1	PLK1 is involved in cell cycle regulation and checkpoint responses to DNA damage; functional outcome of W binding unknown	NiV (63)	Currently no reports
	PRP19 complex	Increases p53 activity in reporter assay; function(s) unknown	NiV (87)	Currently no reports
	STAT1	Sequesters inactive STAT1 in the nucleus, inhibiting Type I IFN signaling	HeV and NiV (46, 65–67)	Yes, rinderpest virus (68)
	Interacts with STAT4	Inhibits STAT4 activity in a reporter gene assay; unknown functional outcome	NiV (71)	Currently no reports
C	Interacts with IKKα	Inhibits TLR7/9-activated induction of IFN-α *via* inhibition of IKKα phosphorylation of IRF7	NiV (41)	Yes, SeV, measles virus, and bovine parainfluenza virus type 3 (41)

^*a*^ Proteomic studies have identified multiple interactors of henipavirus proteins (62, 87); Table 1 shows experimentally characterized/validated interactors implicated in subversion/modulation of host cell biology.

## INTRACELLULAR MODULATION OF HOST BIOLOGY BY HENIPAVIRUS STRUCTURAL PROTEINS

### F and G proteins

F and G proteins are principally involved in cell attachment and entry, but several data indicate broader roles in virus-host interactions. Proteomics analysis of NiV virus-like particles (VLPs) produced by cells expressing F, G, and M proteins identified host proteins potentially incorporated into the virion, including proteins associated with vesicle sorting and transport (88, 89), protein oligomerization (88), and cytoskeletal factors (88, 89)—several of which were found to reduce budding efficiency when experimentally depleted from cells including Vps4A, which is involved in the Endosomal Sorting Complex Required for Transport (ESCRT) pathway (89). This suggests that many intracellular factors may affect virion formation and hence could be alternative targets for antiviral approaches, distinct from virus-receptor interactions involving F and G, which have been extensively assessed as targets and are reviewed elsewhere (90, 91). Indeed, the analysis of NiV G and F interactors via affinity purification (87) and proximity labeling approaches (62) identified various potential cellular interacting proteins, including Serpine mRNA binding protein 1, stathmin 1, and cortactin, with cortactin overexpression inhibiting NiV pseudovirus infection (Table 1) (62). These studies currently lack validation using infectious viruses but may provide a starting point for new potential antiviral targets.

### M protein

**Fig 4:**
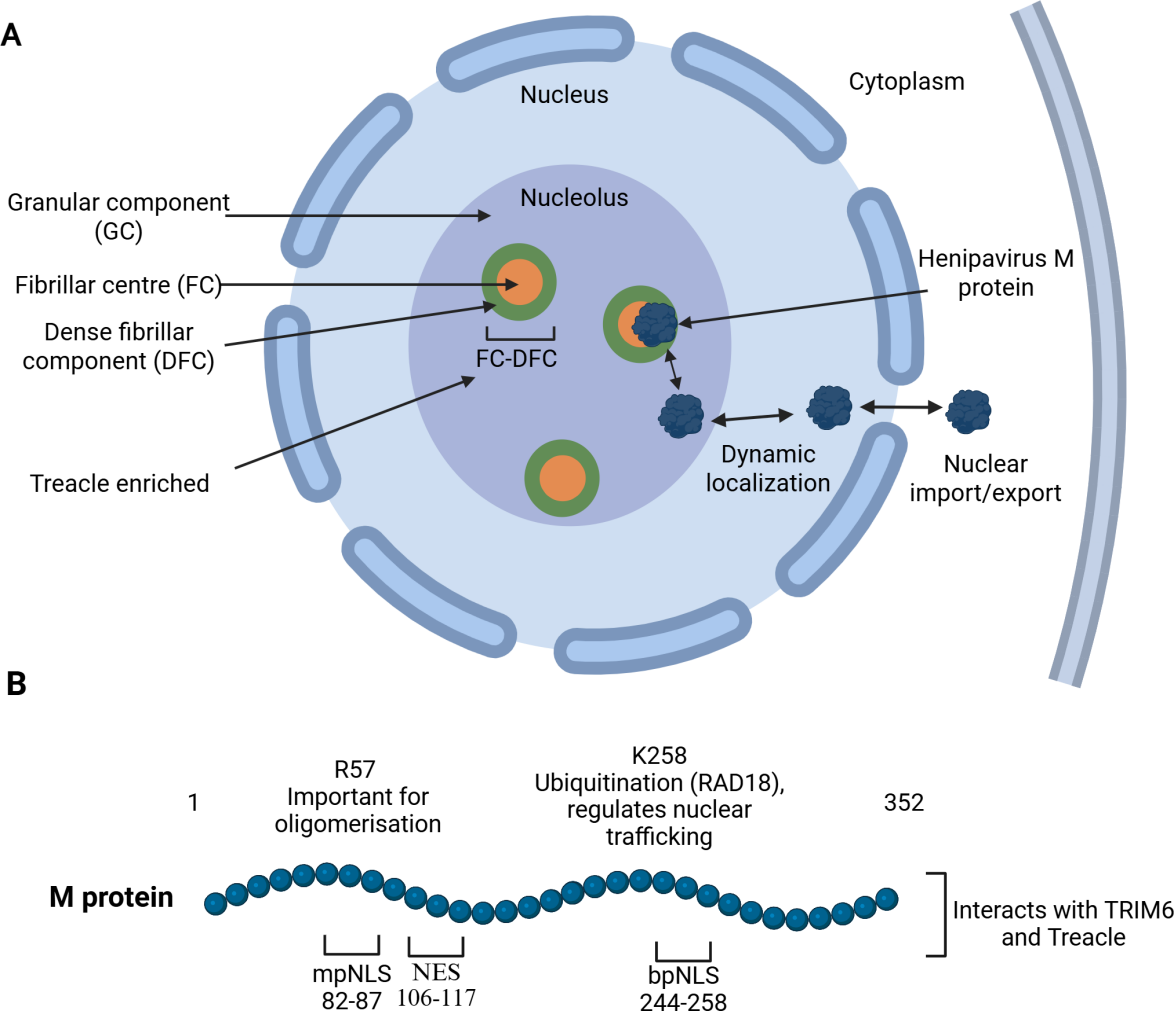
Henipavirus M protein localizes to the nucleus/nucleolus to form specific host protein interfaces. (**A**) Nucleocytoplasmic and nucleolar localization. HeV/NiV M protein can traffic between the cytoplasm, nucleus, and nucleolar compartments, via interactions including IMPα, XPO1, and potentially intranucleolar proteins such as Treacle (which is enriched in FC/DFC) (58), resulting in a dynamic localization (see text). (**B**) Interactors and key residues of HeV/NiV M protein (43). Residue R57 is important for M protein oligomerization (92). M protein also contains a NES and two characterized NLSs enabling nucleocytoplasmic trafficking (52, 57). Ubiquitination of residue K258 in the M protein bpNLS is mediated by RAD18, which facilitates the transfer of ubiquitin from the E2 ubiquitin-conjugating enzyme RAD6A (61); this ubiquitination regulates nuclear shuttling, required for efficient viral egress and host modulation. M protein interacts with Treacle to inhibit rRNA synthesis (58) and TRIM6 to suppress IFN induction (43).

M protein in the virion is located beneath the inner leaflet of the envelope and has roles in coordinating virion assembly and host modulation (Fig. 2) (92, 93). The central role of M protein in budding was identified by analysis of VLP production by cells expressing M protein alone or in combination with F and G proteins (94); furthermore, NiV lacking M protein has altered virion morphology and reduced viral titers, highlighting the importance of M protein to infection (95). Interestingly, although these conserved functions of M protein are located in the cytoplasm/plasma membrane (where all basic replication processes occur), henipavirus M proteins also target the cell nucleus/nucleolus (Fig. 4) (53, 56). Notably, budding by M protein of NiV, HeV, and several other henipaviruses/paramyxoviruses (53, 96) requires nucleocytoplasmic trafficking, involving interactions with host proteins, and post-translational modifications including ubiquitination (52). Nuclear trafficking of NiV M protein is mediated by a conserved bipartite nuclear localization signal (bpNLS) (96) and an auxiliary monopartite NLS (mpNLS) (52, 57), which interact with cellular importin (IMP) proteins that mediate protein trafficking into the nucleus. Structural analysis revealed that the bpNLS uses a non-classical mechanism for nuclear trafficking (binding to the IMPα minor binding site), whereas the mpNLS binds the major site (57). Nuclear NiV M is exported to the cytoplasm by a CRM1/exportin 1 (XPO1)-dependent nuclear export signal (NES) (52), regulated by monoubiquitination of lysine K258 in the bpNLS (Fig. 4) (52).

Although nucleocytoplasmic trafficking and the bpNLS of M proteins appear conserved across the genus (96), and some paramyxoviruses (53), the extent of nuclear localization varies among henipaviruses. Specifically, nuclear localization of M protein of GhV (previously Kumasi virus) is reduced compared with those of NiV, HeV, and CedV M proteins in transfected cells (96), and this correlates with a lower capacity of GhV M protein to form VLPs; a similar but less pronounced trend was observed for CedV M (96). The significance of the inter-species differences remains unclear, but the data generally support the roles of M protein nuclear trafficking in efficient virus budding and may suggest differences in the assembly and replication of different henipaviruses.

The nuclear localization of M protein is implicated in the manipulation of host cell biology, based on the identification of multiple interactions of M protein with nuclear proteins, such as the nuclear acidic leucine-rich nuclear phosphoprotein 32 family member B (ANP32B) for HeV, NiV, and other paramyxoviruses (50, 51). Knockdown of ANP32B did not affect NiV budding, but the interaction may be affecting cellular functions of ANP32B such as gene expression regulation, although this has not been investigated (50). However, the nature/function of many interactions with nuclear proteins is poorly defined, and the most significant insights on specific roles of nuclear localization of M protein appear to relate to interactions with the nucleolus. The nucleolus is a sub-nuclear compartment conventionally known for central roles in ribosome biogenesis, but it also regulates transcription, stress responses, the DNA damage response (DDR), and other cellular processes (97). It is a large, membrane-less organelle comprising at least three immiscible sub-compartments (the fibrillar center [FC], dense fibrillar component [DFC], and granular component [GC]) formed through liquid-liquid phase separation (LLPS) (98), which is driven by weak molecular interactions, often mediated by intrinsically disordered regions (IDRs) or multivalent domains in proteins (99). Increasing evidence suggests the nucleolus is a common target of viruses (100, 101), including those that replicate in the cytoplasm, to modulate host responses. However, specific intranucleolar functions of RNA virus proteins are not well-characterized, with the henipavirus M protein being one of the best studied.

Henipavirus M protein nuclear/nucleolar trafficking appears to be highly dynamic (52, 93, 102), with nucleolus transit reported to be a prerequisite to assembly and budding functions, suggestive of a regulatory role of nucleoli in viral release (52, 93). M protein interactome studies identified multiple nucleolar proteins (53, 56, 58). The first key data indicating the potential importance of nucleolar proteins in henipavirus infection came from a genetic screen, which showed that fibrillarin (FBL) is essential for infection by HeV and several other paramyxoviruses (56). FBL and M protein interact and co-localize within nucleoli; however, the relevant role(s) of FBL during infection remain unclear (56).

The first evidence for the specific intranucleolar function of M protein (58) showed that HeV M accumulates within sub-nucleolar FC-DFC compartments, which are the sites of ribosomal RNA (rRNA) transcription and processing (Fig. 4). HeV M was bound to and colocalized in FC/DFC with the nucleolar protein, Treacle, and Treacle knockdown prevented M protein FC/DFC accumulation. Treacle is crucial for rRNA synthesis/processing and is involved in the nucleolar DDR where it interacts with Nijmegen Breakage Syndrome 1 (NBS1), leading to the inhibition of rRNA synthesis (103). HeV M protein-Treacle interaction similarly inhibits rRNA biogenesis, potentially by mimicking the NBS1-Treacle mechanism that occurs during a DDR. The functional significance remains unclear, but it has been speculated that it may help the virus navigate a hostile host environment, protect infected cells from DNA damage, or facilitate replication in bat cells with high reactive oxygen species levels (104, 105). Alternatively, M protein may impact ribosome production, affecting the translational landscape to modulate host cell biology (58, 59, 104).

Treacle interaction and inhibition of rRNA biogenesis are conserved among NiV, CedV, and MojV, albeit with quantitative differences (59). Intriguingly, mumps virus M protein and rabies virus P3 protein also interact with Treacle, and P3 was shown to impair rRNA biogenesis (59, 60). However, Treacle depletion affected virus production differently, promoting HeV replication while reducing rabies and mumps virus replication (58–60). Thus, targeting Treacle or related pathways may represent a conserved viral strategy, although functional outcomes appear to differ between viruses.

Henipavirus M protein also antagonizes IFN responses (Table 1) by promoting the degradation of the ubiquitin ligase TRIM6 (43), which prevents TRIM6-dependent ubiquitination of IKKε, IRF3 activation, and type-I IFN transcription (Fig. 3). Thus, TRIM6 degradation by M protein suppresses IFN induction, and this function is conserved across henipavirus species (NiV, HeV, GhV, and CedV) (43). Interestingly, mutation of K258 within the bpNLS impaired this function, suggesting nucleo-cytoplasmic trafficking is required (43). Since K258 ubiquitination regulates trafficking, host subversion, and viral budding, it represents a potential antiviral target. This is supported by the observation that depletion of free ubiquitin in cells (using proteasome inhibitors such as MG132 and bortezomib which are FDA-approved for certain cancers) inhibits K258 ubiquitination and reduces NiV titers (52).

### P protein and alternative products of the P gene

#### P gene editing

**Fig 5:**
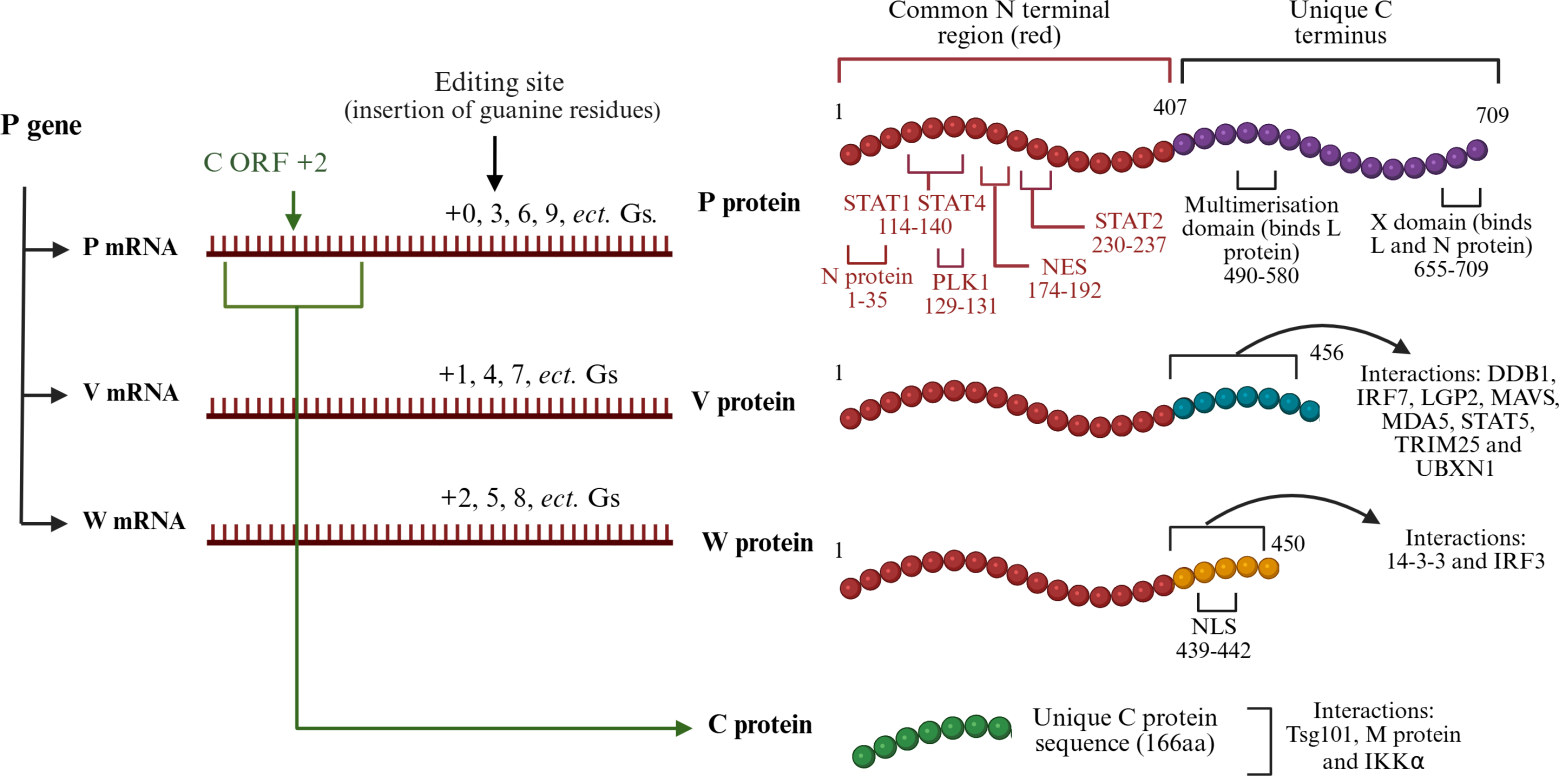
The HeV/NiV P gene encodes multiple proteins through RNA editing and alternate reading frames. The HeV/NiV P gene generates mRNA encoding P protein through faithful transcription, or V or W through the insertion of additional Gs by the viral RdRp at a defined RNA editing site, creating a + 1 (i.e., insertion of 1, 4, 7, etc. Gs) or +2 (insertion of 2, 5, 8, etc. Gs) reading frame, respectively; insertion of multiples of three Gs (3, 6, 9, etc. that maintain the reading frame) result in expression of P protein (107). The altered reading frame downstream of the editing site for V or W protein transcripts results in translation of proteins with a common N-terminal region (NTR) (405 amino acids for HeV and 407 for NiV) and different C-terminal regions (CTR) (65, 74). Known host cell/viral interactors and localization sequences (based on NiV) are shown; those shared between the proteins are shown in red and those that are unique in black (see Table 1 for references). The NES is conserved in P, V, and W but only appears to impact the localization of V and W (74). P protein binds to L and N via its unique multimerization domain and X domain as shown (106, 108), and additionally binds N via residues in the NTR (106) (it has not been reported if these residues in V and W also bind N). Translation of P mRNA from an internal alternate start codon (using a + 2 reading frame within the region encoding the P/V/W NTR) produces C protein (23).

Paramyxovirus P genes encode the conserved P protein, produced by most paramyxoviruses through faithful transcription of the P gene ORF. It is the essential co-factor for the polymerase, L protein, for genome transcription and replication, and binds N protein as a chaperone, preventing N polymerization and nonspecific N-RNA binding (106). The P gene also encodes several accessory proteins which can include V, W, and C (23). In common with other paramyxovirus genera, V and W transcripts of henipaviruses are generated via editing of mRNA (addition of guanine residues) at a defined site during P gene transcription, whereas C is translated from alternative reading frame(s) (23, 107) (Fig. 5). Consequently, P, V, and W proteins share a common N-terminal region (NTR), and each protein has a unique C-terminal region (CTR) (23, 65), whereas C protein is unrelated in sequence (23). Most henipaviruses/parahenipaviruses, with the exception of CedV, appear to contain the P gene editing site and so would be expected to be able to produce V and W (7, 14); however, only HeV/NiV have been shown to generate these proteins (107).

### Functions of P gene products

P gene-encoded proteins appear to form a major part of the intracellular virus-host interface through interactions with host proteins that mediate functions distinct from basic processes in viral replication, assembly, or structure (below) (87). The principal conserved function of P protein is in genome transcription/replication, but P protein also mediates immune evasion mechanisms. C, V, and W appear to function primarily in immune evasion as the L- and N-binding regions in the unique CTR of P are lost; however, it is unknown if V and W bind to N protein via a secondary binding site in the common NTR, and hence, these proteins may have some role in replication/transcription (Fig. 5) (106). Indeed, potential roles of V, W, and C proteins in regulating transcription/replication have been indicated, based on inhibitory effects of expression in a minigenome system (109).

### Roles of alternative P gene products in virulence

#### V and W proteins

Although P protein is critical for viral replication, alternative functions of P protein and accessory P gene products in processes such as immune evasion are expected to contribute to virulence. Critical replication functions of P protein preclude the analysis of viable virus deleted of P protein, but V and W protein-deficient recombinant viruses have been generated by reverse genetics, enabling assessment of roles of these proteins in infection, including *in vivo* (110, 111). Infection by V-deficient NiV in ferret and hamster models was non-lethal compared with lethal wild-type (WT) virus (110, 111), consistent with multiple roles as an antagonist of innate immune responses (below, Fig. 3 and 5). In contrast, W protein knockout was not associated with reduced lethality in hamsters (110, 111), with no clear differences in disease progression/histopathology between W-deficient and WT NiV (111). However, in a ferret model (which more accurately models aspects of NiV disease in humans [112]), infection by W-deficient virus still had lethal outcomes, but with differences in disease progression (decreased respiratory disease severity, stronger cytokine response, increased neurological disease severity) (110). Elevated cytokine levels and/or prolonged disease duration may facilitate the spread of W-deficient NiV to the brain, indicating that W protein modulates the host inflammatory response and influences disease course.

Further indications of the potential importance of V and W proteins in disease come from CedV, which appears to lack the P gene mRNA editing site and therefore the ability produce either protein, and shows no clinical signs of disease in infected ferrets, hamsters, or guinea pigs (113, 114). The hosts appear to be able to mount an efficient IFN response to clear CedV, consistent with the lack or greatly reduced V/W protein (114, 115) and indicating important differences in the intracellular host interface of different henipaviruses. Together, these data underscore the potential of V, and potentially W, proteins as therapeutic targets.

#### C proteins

There are conflicting findings on the effect of NiV C protein knockout. In hamsters, C knockout significantly reduced lethality, similar to the V knockout (111, 116), with increased inflammation/cytokine expression compared with WT NiV, consistent with the roles of C protein in regulating immune responses (116) (below, Fig. 3 and 5). However, in ferrets, C deletion did not prevent a lethal outcome (117) but reduced respiratory disease severity (possibly relating to roles in regulating innate immunity), similar to the W knockout, except that neurological disease was comparable with that for WT NiV (117). These findings highlight that various factors contribute to disease outcomes and the different impacts of the V, W, and C proteins. This is consistent with differences in their specific functions (discussed below), providing rationale for the evolution and maintenance of multiple alternative, overprinted products in a single gene, despite broadly similar roles such as in immune evasion.

### Shared functions of P, V, and W proteins

The common P/V/W NTR of NiV is largely intrinsically disordered, with some transient alpha helices, including in the first 50 residues (106, 118). Additionally, a region encompassing residues 200–310 forms amyloid-like fibrils in NiV/HeV proteins, with three contiguous tyrosine residues that are important to promote phase separation (119, 120). This property may be important to the formation of inclusion bodies (IBs), which are induced in virus-infected cells, contain high levels of viral RNA, P, and N proteins and can sequester host proteins (121), thereby potentially aiding immune evasion by P protein.

The flexibility of IDRs enables interactions with multiple partners. The P/V/W NTR binds and prevents the phosphorylation of STAT1 (NiV residues 114–140 [46]) and STAT2 (NiV residues 230–237 [66]) (Fig. 3), with STAT1/2 targeting being a common strategy among paramyxoviruses (122) (Table 1). In reporter assays, NiV V and W show stronger inhibition of IFN-dependent STAT1/2 signaling than P protein, consistent with more specialized functions in immune evasion (65). CedV has a compromised ability to inhibit STAT1/2 signaling compared with HeV, likely due to the absence of or reduced V and W proteins and substitutions between HeV and CedV of residues implicated in P protein-STAT1 binding (67).

It has been proposed that NiV sequesters inactive STAT1 in the nucleus via the W protein, which exhibits strong nuclear localization compared to the cytoplasmic P and V proteins (46). STAT1-GFP was also nuclear during WT NiV infection, and this localization was disrupted in cells infected by NiV with mutations preventing STAT1 binding. This indicates that W protein-STAT1 binding in the nucleus is the primary mechanism of STAT1 antagonism in infected cells (46). However, another report indicated that endogenous STAT1/2 is cytoplasmic in NiV-infected cells and co-localizes with NiV N protein in IBs, suggesting that P and N proteins can sequester STAT1/2 in cytoplasmic IBs, representing an additional potential antagonistic mechanism for P protein (121). Differences in the experimental design of these studies (e.g. analysis of overexpressed vs. endogenous STAT1, cell type, and transfection vs. infection to examine IFN signaling) may explain the differing observations. Future assays using recombinant live virus infection and combinations of different cell/tissue models may provide clearer insights into the relative contribution of different P gene products to STAT antagonism (121).

NiV with the mutation Y116E in P/V/W (which prevents STAT1 interaction) (46) remains lethal in ferrets, albeit with a modified disease course (123). The infection resulted in extended survival time, fewer lesions in several organs, and increased neurological disease, similar to W- or C-deficient NiV. The increased neurological disease may be due to the prolonged lifespan of infected animals or potentially increased JAK/STAT signaling in neuronal cells infected by the mutant viruses. Overall, the lethality of the NiV Y166E mutant underscores that STAT1 antagonism by P/V/W is only one aspect of the viral infection/immune evasion/virulence mechanisms, where multiple processes (for example, N protein inhibition of STAT1/2 [45]) form a multipronged strategy for efficient inhibition of the IFN response.

The NiV P/V/W NTR also targets STAT4 (71), which mediates signaling by an array of cytokines (including IFNs), and other stimuli (44, 124). Mutation of NTR residues 114–140 (Fig. 5) impairs STAT4 (and STAT1) inhibition (46, 71). The importance of STAT4 targeting has not been fully elucidated as STAT4 is involved in a range of signaling pathways (124), but it may relate to IFN, as some proinflammatory roles of STAT4 are activated by IFNα (125). HeV/NiV thus appear to affect not only broad but also selective targeting of signaling by IFN (and possibly other cytokines) through STAT1, 2, and 4, but not STAT3 or STAT6 (71), possibly reflecting specific outcomes conducive to henipavirus infection (44, 71).

Finally, henipavirus P/V/W proteins (shown for NiV) bind to Polo-like kinase 1 (PLK1) (63), which has key roles in several cellular processes including regulation of the cell cycle, the DNA damage response, and innate immunity (126, 127). Both HeV and NiV V proteins can be phosphorylated by PLK1, with residues 199–201 in HeV and 129–131 in NiV being critical for this interaction (63). PLK1 interaction did not appear to affect replication in a minigenome assay; hence, roles of the interaction/V phosphorylation remain unresolved, although the non-replication accessory functions of V protein suggest effects will be at virus-host interfaces, potentially in immune evasion (63).

### Subcellular localization of P-gene products

HeV/NiV P, V, and W proteins differ in subcellular localization, primarily due to differing trafficking sequences in their unique CTRs and shared sequences in the NTR; specifically, P/V are cytoplasmic, whereas W is largely nuclear (65). Leptomycin B (LMB, an XPO1 inhibitor) increases nuclear localization of V and W, but not P protein (74), suggesting that localization of V/W is dependent on XPO1-mediated nuclear export, involving a NES in the NTR (within residues 174–192 in HeV P/V/W). The common NTR also interacts with IMPα1 and so appears to contain an NLS (74), although specific residues have not been mapped (74). This indicates that V and W can undergo nucleocytoplasmic shuttling through IMPα1/XPO1 interactions (74), and this presumably enables interactions with nuclear proteins (which are otherwise separated from the cytoplasm where viral replication occurs), and/or interference with trafficking of cellular factors, as reported for Ebola protein VP24 (128) and Middle Eastern Respiratory Syndrome (MERS) protein 4b (129). The lack of effects of LMB on P protein suggests that it does not shuttle to the nucleus, likely due to the lack of significant NLS activity; as P protein is 78 kDa in size (exceeding the size permitting diffusion through the nuclear pore), it would require active transport via IMPs or will remain in the cytoplasm. The differences in P protein compared with V/W may relate to effects on trafficking signals due to protein conformation, post-translational modifications, or potential interactions with other proteins resulting in masking of sequences or sequestration.

Notably, although V is predominantly cytoplasmic at steady state, W protein is nuclear, due to an additional strong NLS in its unique CTR (Fig. 5) (84). The precise roles of differing localization of P, V, and W are not resolved, but data indicate the importance of immune evasion (below). Of interest, inhibition of XPO1-mediated nuclear export by LMB, or of nuclear import by ivermectin, reduced HeV titers (74). This is consistent with the importance of viral protein trafficking (74) and its potential to provide targets for antiviral drug design; however, the broad effect of these treatments on trafficking, including of cellular proteins (e.g., transcription factors, immune signaling molecules, etc*.*) and possibly other cellular processes, means that further research is required to define the underlying mechanisms.

Taken together, the current data indicate that the common NTR (residues 114–236 in particular) bind multiple host proteins including STAT1/2/4, XPO1 (for V/W), and PLK1 (63). This region may thus provide targets for antiviral approaches that could concurrently inhibit multiple functions of P, V, and W (74).

#### P gene products and IBs

In virus-infected cells, P protein accumulates with cytoplasmic IBs, where it colocalizes with N protein (130). N protein binds the RNA genome forming the nucleocapsid and the RdRp (via P protein) to facilitate viral transcription and replication (22, 106).

Many mononegaviruses form cytoplasmic IBs, which are typically membrane-less compartments formed by LLPS that contain large amounts of viral nucleocapsid (131). P and N proteins comprise the minimal components required to form IBs, with M protein present in NiV IBs located near the plasma membrane, in contrast to NiV IBs in the cytoplasm/peri-nuclear region (130). Mononegavirus IBs are often considered to be viral factories, where high levels of viral mRNA synthesis and genome replication are localized (131). However, for several viruses including henipaviruses, this role has not been conclusively confirmed, and other functions at the virus-host interface have been identified. First, IBs may have aggresome-like properties to prevent proteotoxic stress; aggresomes are cellular structures that are aggregations of misfolded proteins, formed under conditions including stress and viral infection (132). The production of large amounts of viral proteins can lead to the accumulation of misfolded proteins, resulting in proteotoxic stress and cytotoxicity. However, in NiV-infected cells, aggresomes do not appear to form, and instead, viral IBs appear to partially fulfill their roles, sequestering viral proteins and other non-essential proteins to limit proteotoxic stress pathways (133). IBs were also recently proposed to act as sites for sequestration of immune proteins (STAT1/2) (121), likely via interactions with P protein, which has long-established roles in STAT1/2 antagonism (46, 65). N protein is also likely involved, as it binds and inhibits STAT1/2 nuclear localization when expressed alone (Table 1) (45). However, the precise contributions of P and N proteins within IBs and the involvement of other viral proteins (e.g., STAT-binding V/W) or additional immune/host proteins remain unresolved.

### Specialist functions of V protein

V protein shares immune evasion functions with P and W proteins due to the common NTR but has additional roles conferred by its unique cysteine-rich CTR (Table 1) (Fig. 5), including regulating multiple elements of the IFN induction pathway via CTR interactions with the PRRs LGP2, RIG-I, and MDA5, and PRR activators TRIM25 and PP1α/γ (Fig. 3) (33, 34, 37, 38, 75). These mechanisms appear to be conserved across V proteins of other assessed paramyxoviruses (33, 34, 37, 38, 75). The V CTR binds MDA5 and LGP2 via their helicase domains that mediate RNA recognition (32, 134). Although early studies using reporter gene-based assays failed to detect inhibition of RIG-I signaling by paramyxovirus V proteins (including HeV/NiV) or V-RIG-I interactions by immunoprecipitation (32, 135), a more recent study reported interactions between the RIG-I CARD domain and V proteins of NiV and other paramyxoviruses including measles virus and Sendai virus (SeV) (Table 1) (33). Activation of RIG-I involves modification of the CARD by TRIM25-mediated ubiquitination, and V protein also directly binds to TRIM25 (33). V protein binding to RIG-I and TRIM25 inhibited RIG-I ubiquitination and binding downstream to MAVS, thus inhibiting the induction of IFN expression. The NiV V CTR also binds and stabilizes UBXN1, a negative regulator of MAVS in response to RLR signaling; this augments the inhibitory effects of NiV V protein-MDA5 interaction (36).

V proteins of NiV, HeV, and several other paramyxoviruses including Newcastle disease virus (NDV), target MAVS and other adapter proteins downstream of RLRs (Table 1) (39). Protein transfection assays indicated that increasing concentrations of V proteins in cells correlate with reduced levels of MAVS, and NDV V protein was found to interact with MAVS directly, causing ubiquitin-mediated degradation (39). V proteins of numerous paramyxoviruses, including NiV, also bind to IRF7 to inhibit its transcriptional activity, which is required for IFN induction in response to several PRRs, including TLR7/9 (40). Thus, the V protein CTR suppresses IFN production via several mechanisms, presumably to effect highly potent antagonism of activation of the antiviral IFN system and complement further mechanisms to prevent subsequent signaling by STATs (see above). V protein also appears to have additional unique STAT-inhibitory mechanisms, including binding STAT5, dependent on residues in the V CTR (71). As STAT5 is implicated in the antiviral IFN response (136) as well as signaling by various other cytokines and growth factors including IL-2 and prolactin, this potentially extends the effects of V protein across IFN/cytokine signaling pathways (44); the functional outcomes of this interaction, however, remain undefined. HeV and NiV V protein also binds to DNA damage binding protein 1 (DDB1, a component of the ubiquitin ligase E3 complex), dependent on the zinc finger domain (ZnFD) of the V CTR (72). V proteins of several non-henipavirus paramyxoviruses bind to DDB1/other components of the ubiquitin ligase complex to enable the degradation of V protein-associated STAT proteins (48, 137, 138). Thus, DDB1 binding may provide an additional mechanism of IFN/STAT antagonism by henipavirus V protein that is not shared with P and W.

Finally, the NiV V protein can inhibit the NLRP3 inflammasome, which consists of the NLRP3 sensor (NOD-, LRR-, and pyrin domain-containing protein 3), ASC adaptor, and caspase-1 effector (139). NLRP3 activation by stimuli including pathogenic RNA and ATP leads to the release of the pro-inflammatory cytokine IL-1β and triggers pyroptosis, an inflammatory form of cell death (139). NiV V, but not NiV P, prevents NLRP3-dependent IL-1β release (77), indicating the importance of the unique V CTR. Binding of the V protein to NLRP3 inhibits NLRP3 self-oligomerization, which is important for inflammasome assembly (77).

### Specialist functions of W protein

The main distinguishing property of W protein compared with V and P protein is its nuclear accumulation (84), which enables interaction/interference with host nuclear proteins and appears to be important for specific mechanisms of IFN antagonism (reviewed [140]). For example, dependent on its NLS/nuclear localization, NiV W can reduce levels of active phosphorylated IRF3 by a currently undefined mechanism, resulting in decreased IFN-β transcription (42). W protein also binds non-phosphorylated STAT1 in the nucleus, which appears to inhibit STAT1 activation by preventing interaction with IFNAR/JAKs at the plasma membrane (46). Furthermore, although the P, V, and W proteins all bind to the PRP19 complex, nuclear localization of W protein allows it to inhibit the p53 repressor activity of PRP19 (87), such that W protein increased p53 activity in a reporter assay. This may suggest modulation of the apoptotic response; however, the PRP19 complex has many cellular roles indicating other outcomes may be important (87).

The HeV W protein CTR also interacts with members of the 14-3-3 protein family (82). 14-3-3 proteins bind to phosphorylated proteins to regulate a range of cellular functions including metabolism, extracellular matrix organization, and apoptosis, such that targeting by W protein provides the potential to modulate multiple processes (82). Importantly, the W protein/14-3-3 interaction is thought to enable the inhibition of transcription factors, including NF-κB, downregulating immune gene expression (82, 140) and dampening inflammatory responses (83). NF-κB inhibition is dependent on W protein nuclear localization and attributed to a resulting nuclear accumulation of 14-3-3 that increases negative feedback of the NF-κB pathway (83). These data for the 14-3-3 and PRP19 complex (87) suggest that W protein may be regulating expression from a range of host genes.

### C protein

Henipavirus C protein is implicated in regulating viral replication, budding and IFN antagonism (141). In common with W and V, NiV C protein can shuttle between the nucleus and cytoplasm, highlighting roles for trafficking in virus-host interactions of accessory proteins (142). Nevertheless, C protein is predominantly cytoplasmic at steady state, and its known IFN antagonist function occurs principally in the cytoplasm (107, 141).

C protein sequences can differ significantly between paramyxoviruses, as C proteins are classified solely as proteins encoded in alternative ORFs in the P gene; indeed, some viral species encode multiple C proteins (141). Nevertheless, C proteins typically share certain features, including a basic character, the presence of IDRs in the CTR, alpha helices in the NTR, roles in regulating viral transcription/replication, and nucleocytoplasmic shuttling activity (141). Although critical residues have been identified for NiV C protein nuclear import and export, these do not resemble classical NES/NLS sequences. Moreover, NiV C protein localization was not altered by XPO1 inhibition, suggesting the use of alternative nuclear export pathways/mechanisms (142).

Regulatory roles of NiV C protein in viral replication were suggested by inhibitory effects of expression in a minigenome replication assay (109). However, NiV lacking C protein produces lower viral titers than the WT virus (46), indicating that C protein facilitates infection by the authentic virus. This is possibly due to regulated/temporal roles in genome transcription/replication or other functions such as in virus budding (which is not assessed in a minigenome assay) and immune evasion. For example, NiV C protein interacts with M protein (which has key roles in viral budding) and Tsg101, an essential factor in the ESCRT pathway. ESCRT catalyzes membrane scission and, hence, is exploited for viral budding. NiV titers were reduced when Tsg101 was depleted from cells, highlighting its importance to infection; thus, C protein deficiency may result in reduced production of extracellular virions (143).

Similar to V protein, NiV C protein inhibits IRF7 phosphorylation but achieves this through interaction with IKKα, rather than IRF7 (Fig. 3) (41); this enables C protein to prevent TLR7/9-activated induction of IFN-α in plasmacytoid dendritic cells. These additional levels of antagonism of pathways that are also targeted by V and W protein (above) may enable highly potent antagonism or potentially have differing impacts in different tissues or stages of infection; such considerations may be relevant to the differing disease outcomes associated with knockouts of the different proteins.

A recent study also reported that a peptide analog of the HeV C protein activates the NLRP3 inflammasome, which appears to contribute to a detrimental inflammatory response in mice (144). This contrasts with the NLRP3-inhibitory function of NiV V protein (77) and potentially represents a mechanism by which the host can trigger innate immunity via NLRP3-dependent detection of viral proteins that would otherwise suppress the immune response. This possibly forms an intricate feedback loop in the evolutionary “Red Queen’s Race” between the host immune response and viral immune evasion (145, 146).

## CONCLUSIONS

HeV and NiV have been extensively researched since their emergence in highly lethal disease outbreaks. This has revealed the multifunctional M, P, V, W, and C proteins as key components in forming the intracellular virus-host interface, whereby viruses subvert host cell processes. Recent research has significantly advanced understanding of the functions/interactions of these proteins in modulating processes including immunity, inflammation, metabolism, nucleolar functions, cell death, and disease pathology. However, the mechanisms and significance of many identified functions/interactions of these proteins remain only partially understood, including the roles of W protein in modulating host regulatory molecules (e.g., 14-3-3), role(s) of N/P protein-dependent IBs in infection, and functional outcomes of M protein regulation of elements of the nucleolar DDR. Additionally, most studies of henipavirus protein functions have focused on single proteins or minimal systems (e.g., minigenomes) rather than infectious viruses, due to requirements for Biosafety level 4 containment for HeV/NiV research. Contrasting results from experiments using isolated proteins compared with infection, and between different studies using viral infection, indicate the importance of validating results between different systems (e.g., cells, tissues, and animals) to clearly define viral protein functions.

Despite some studies encompassing multiple members of the henipavirus genus (4, 59), the conservation or divergence beyond HeV and NiV of many of the functions/interactions of viral proteins (especially the P gene products) is poorly defined. This is significant given the discovery of additional genotypes of HeV/NiV and new species of henipaviruses/parahenipaviruses, including viruses that have spilled over into human populations (15, 147). A deeper understanding of the conservation/divergence of protein structure and function should contribute to the development of drugs or vaccines to minimize future outbreaks, including the development of approaches that may have broad anti-henipavirus activity. This knowledge will also aid in understanding the risks posed by outbreaks of different viruses, including how differences in virus-host intracellular interactors can affect pathogenesis.

A recent review of potential drug candidates for NiV (148) highlighted that research has focused on F and G proteins, due to roles in viral fusion/attachment, and the successful application of such approaches for other viruses (e.g., SARS-CoV-2 [149]). However, the important roles of other viral proteins in NiV infection, including those forming intracellular interactions with diverse host proteins, provide alternative drug targets. Furthermore, a recent *in silico* analysis identified M protein as a potential vaccine target based on factors such as antigenicity and allergen potential, expanding its possible therapeutic value (150). The identification of significant roles of V protein in pathogenesis and the fact that P/V/W share common regions and functions (and so may be amenable to simultaneous multi-pronged targeting by single antiviral compounds) highlight its potential as a target that may impact multiple infection processes. Although there are currently no approved human vaccines or therapeutics for HeV or NiV, countermeasures are under development (11, 151), and the identification of conserved targets for antiviral development may provide further options.
